# Postoperative wound infection by nontuberculous mycobacteria; case series in Dhaka Medical College Hospital of Bangladesh

**DOI:** 10.1002/ccr3.8264

**Published:** 2023-11-27

**Authors:** Kakali Halder, Nusrat Noor Tanni, Rubaiya Binte Kabir, Maherun Nesa, Md. Faizur Rahman, Rizwana Zaman, Farjana Binte Habib, Noor‐E‐Jannat Tania, Md. Asaduzzaman, Azmeri Haque, Akteruzzaman Chowdhury, Avizit Sarker, Nadira Akter, Mahbuba Chowdhury, Sazzad Bin Shahid, S. M. Shamsuzzaman

**Affiliations:** ^1^ Department of Microbiology Dhaka Medical College Dhaka Bangladesh

**Keywords:** acid‐fast bacilli (AFB), Bangladesh, Dhaka Medical College Hospital, nontuberculous mycobacteria (NTM), postoperative wound infection

## Abstract

The incidence of nontuberculous mycobacterial (NTM) infections after operations is increasing in Bangladesh but data regarding clinical presentation, diagnosis, treatment, and prognosis after treatment are lacking. In this case series, three patients having persistent serous discharge from incision wound after operation were studied. Discharge from wounds were collected, wet film microscopy was performed for pus cells and fungus, Gram stain, Ziehl‐Neelsen (ZN) stain, culture in routine culture media and Lowenstein‐Jensen (LJ) media, Xene‐Xpert for mycobacterium tuberculosis (MTB), polymerase chain reaction (PCR) for NTM were done. NTM‐positive patients were treated initially for 6 weeks with four drugs regimen (clarithromycin 500 mg 12 hourly, ciprofloxacin 500 mg 12 hourly, linezolid 400 mg 12 hourly, and amikacin 500 mg 12 hourly), followed by 5 months with three drugs regimen (clarithromycin 500 mg 12 hourly, ciprofloxacin 500 mg 12 hourly, and linezolid 400 mg 12 hourly) as a maintenance dose. Cessation of discharge occurred within 3–4 weeks after starting treatment, and the wounds were healed.

## Graphical abstract

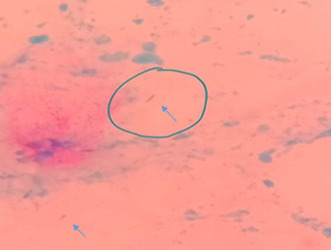

## 1 INTRODUCTION

Nontuberculous Mycobacteria (NTM) are ubiquitous group of bacteria that include mycobacterial species other than *Mycobacterium tuberculosis* complex and *Mycobacterium leprae*.^1^ NTM are the diverse group of organisms that are isolated from environmental sources like soil, water, dust, lakes, rivers, streams and also from municipal water sources like water that people drink or shower.^2^ *Mycobacterium chelonae*, *Mycobacterium abscessus*, *Mycobacterium fortuitum*, and *Mycobacterium smegmatis* are most often linked with NTM infections after surgical intervention worldwide.^3^ Usually, Ziehl‐Neelsen (Z‐N) staining and mycobacterial cultures are not routinely performed; that is why the detection of NTM is missed and the burden is increasing.^4^ Resistance to common antiseptics and disinfectant solutions used in hospital settings is also making these illnesses a growing threat. Inaccurate sterilization of instruments used in operation theater (OT) is usually responsible for NTM infections and makes it a great problem mainly affecting developing countries.^5^ So, proper sterilization of the instruments is essential to prevent the occurrence of postoperative wound infections with atypical Mycobacterium. NTM have a lipid‐rich outer membrane with hydrophobicity and can produce biofilms which make collections of microorganisms that stick to each other, adhere to the surfaces of moist environments, become resistant to antibiotics, are difficult to eliminate, and ultimately increase the likelihood of chronic infection.^3^, ^6^ Globally, in the last few years, nosocomial postoperative wound infections by NTM have been increasing.^7^ Early detection is hampered and may be due to difficulties of identification, and long‐term medications with lowered tolerance make it more challenging to treat effectively.^7^, ^8^ Since treatment differs from species to species, species and subvariants should be identified and recognized from environmental NTM.^9^

In Bangladesh, as a *Mycobacterium tuberculosis* (M.TB) endemic zone, NTM infection is often under‐detected, because it can mimic M.TB. In this case series, several cases having persistent chronic discharges from incision wound for several weeks to several months after operation were analysed regarding clinical presentation, diagnosis, treatment and prognosis of NTM infections.

## 2 MATERIALS METHODS

We collected data on such infections from February 2021 to July 2022 from the patients who presented with complaints of chronic serous discharge from postoperative wounds for at least more than 2 months even after taking several antibiotics for a long time. However, not all the patients from various departments of DMCH, with the same criteria were referred to the microbiology department for testing. Therefore, it is not possible to detect the actual prevalence rate of NTM infection in DMCH.

The cases were examined, detailed history was taken, and wound discharge was collected for microbiological laboratory testing at the microbiology department of Dhaka Medical College, Bangladesh. To detect NTM from these cases, Gram stain, Zeihl‐Neelsen (Z‐N) stain, fungal microscopy of the samples, culture, Gene‐Xpert, and polymerase chain reaction (PCR) tests were done. We identified three patients with postoperative skin and soft tissue infections caused by NTM.

In the microbiology department, with proper aseptic technique, discharge was collected by sterile swab sticks from all the cases. Wet‐film preparation for fungal microscopy, Gram stain, Z‐N stain, Gene‐Xpert, culture, and PCR were done from wound discharge. Discharge was inoculated and incubated on blood agar media and MacConkey agar media for 7 days, and Lowenstein Jensen (LJ) media for up to 6 weeks at 37°C aerobically. Written informed consent was taken from each patient.

Detailed information about the operation history, the treatment regimens of antibiotics, duration of therapy that is completed or running, follow‐up, and the ultimate outcome were collected from patients. In other cases, where NTM were suspected but not identified from the samples, were treated with another treatment protocol advised by the microbiology department of Dhaka Medical College.

## 3 CASE SERIES

Two males and one female, between the ages of 30 and 36 years, who underwent various surgeries presented with serous discharge at the site of incision mostly within a few months of the surgical procedures, which progressed to chronic discharging sinus with a small opening. Most of the patients had a history of apparently healthy postoperative wounds, and stitches were removed within 7–10 days after surgery. The discharge from the wounds was thin, serous, and non‐purulent. Most of them did not give any history of high fever, pain, or any constitutional symptoms associated with the wound discharge, but some had low‐grade fever and mild pain by giving pressure on the wound site.

One case presented with nodular swellings that progressed to chronic discharging sinus from a small site over the incision site. On applying local pressure, the discharge from the sinus of the wound site increased. Those patients gave a history of taking several antibiotics such as fluoroquinolones, colistin, cefixime, vancomycin, ceftriaxone, meropenem, and linezolid previously, but none was cured. One patient took anti‐MTB drug regimens for several months without any improvement and could not show any diagnostic evidence for MTB. Detailed clinical profiles of study cases are shown in Table 1, and investigation profiles are given in Table 2.

**TABLE 1:** Clinical profile of the study cases.

Cases	Age (years)	Sex	Ward	Type of operation	Underlying diseases	Discharge type and duration	History of mycobacterium tuberculosis treatment& duration	History of surgical intervention
1.	35	M	Neurosurgery	Craniotomy	–	Clear, non‐purulent, serous; 1 month	–	–
2.	36	F	Neurosurgery	Middle cerebral artery (MCA) aneurysm	–	Serous, non‐purulent; 13 months	–	Yes
3.	30	M	Neurosurgery	Surgery in the ventral column	–	Serous, non‐purulent; 8 months	Yes, 6 months	Yes

**TABLE 2:** Investigations and treatment profile of the study cases.

Cases	Case 1	Case 2	Case 3
Sample	Wound swab	Wound swab	Wound swab
Fungal microscopy	–ve	–ve	–ve
Gram stain	No pus cells or bacteria	No pus cells or bacteria	No pus cells or bacteria
Z‐N stain	Moderate number AFB	A few AFB	Moderate number AFB
Histopathology	Not done	Not done	Not done
Culture	MRSA, NTM	–	NTM
Gene‐Xpert	MTB not detected	MTB not detected	MTB not detected
PCR	Not done	NTM detected	NTM detected
Treatment	Anti‐NTM regimen, 6 months	Anti‐NTM regimen, 6 months	Anti‐NTM regimen, 6 months
Outcome	Cured	Cured	Cured

Abbreviations: AFB, acid‐fast bacilli; MRSA, methicillin‐resistant *Staphylococcus aureus*; MTB, mycobacterium tuberculosis; NTM: nontuberculous mycobacteria; PCR, polymerase chain reaction; Z‐N stain, Ziehl‐Neelsen stain.

**FIGURE 1:**
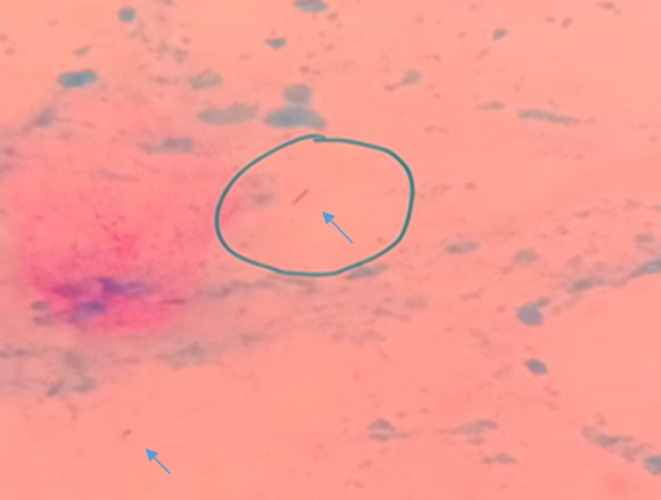
High‐magnification microscopic picture showing the presence of acid‐fast bacilli in wound discharge on Ziehl‐Neelsen stain (100×).

On Gram staining, in some cases, few to moderate amounts of pus cells and sometimes gram‐positive cocci were seen under the microscope. Z‐N‐stained smear revealed acid‐fast bacilli (AFB) in all the cases (Figure 1). No fungal element was found in any sample on microscopic examination of the discharge. No *Mycobacterium tuberculosis* was detected in Gene‐Xpert for MTB from any case.

**FIGURE 2:**
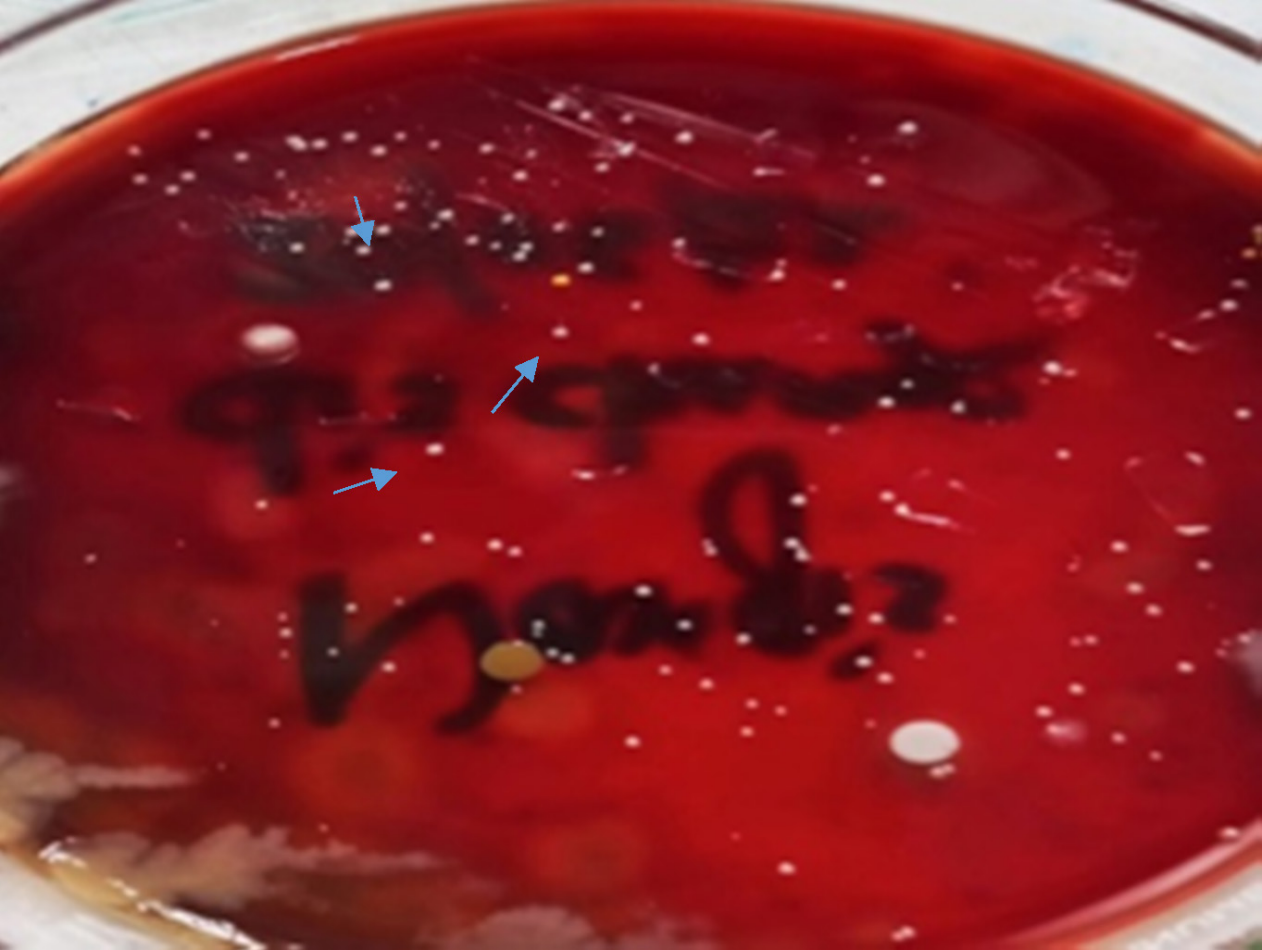
Culture on blood agar media yielded growth of NTM on Day 4.

**FIGURE 3:**
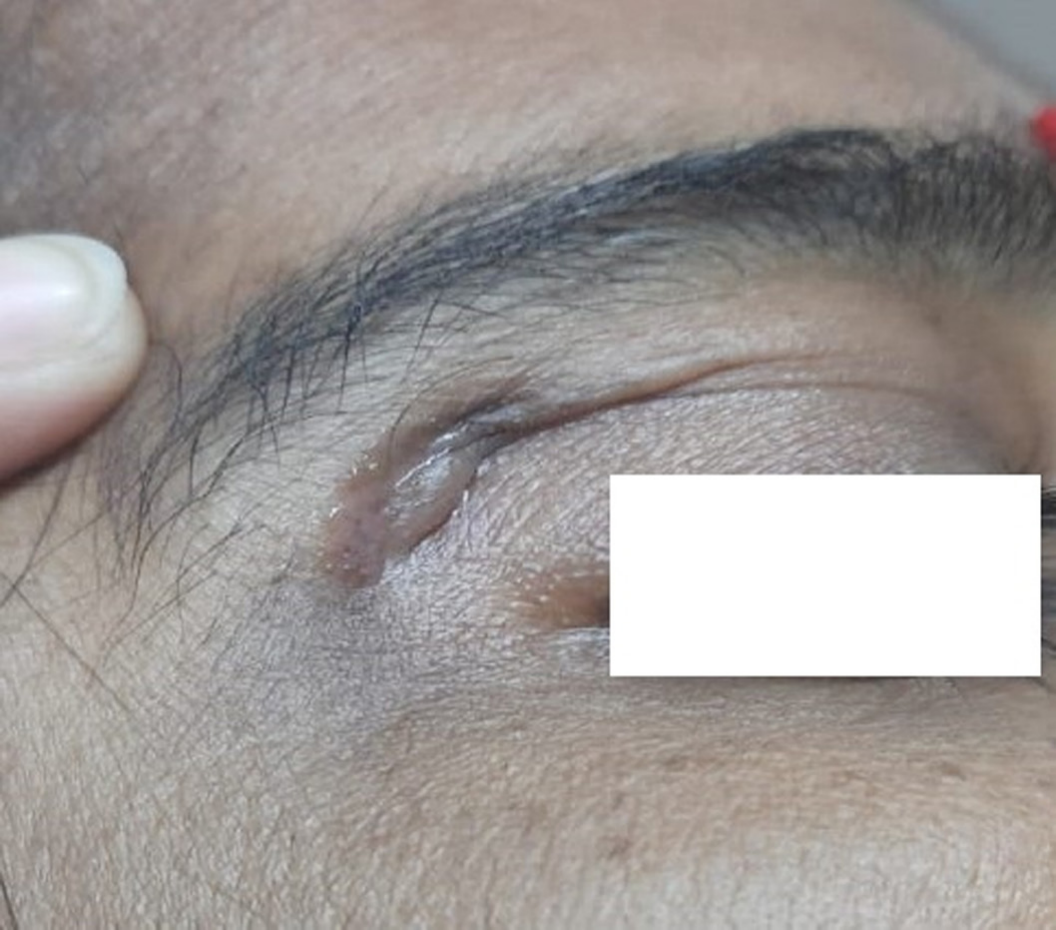
Discharging sinus 3 months after operation when the patient came to the microbiology department.

**FIGURE 4:**
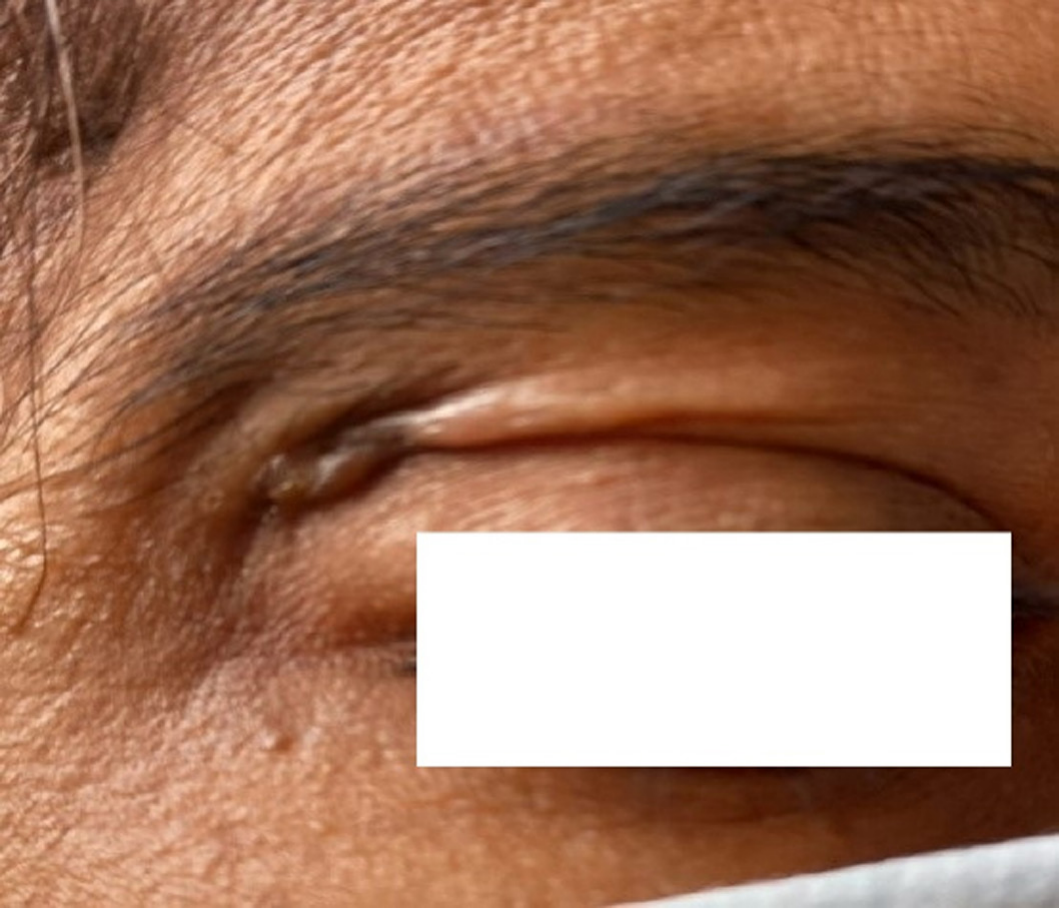
Healed lesion after 6 months of getting treatment.

Culture on LJ media and MacConkey agar media did not show any growth, but on blood agar media, pale and opaque colored colonies were found in two samples after 4 days of incubation at 37°C (Figure 2) which became yellow‐pink after 7 days of incubation. Again, Z‐N stain was done from culture isolates and revealed AFB. PCR was done, and NTM was detected in two specimens. In one sample, culture yielded additional growth of methicillin‐resistant *Staphylococcus aureus* (MRSA) on blood agar media after 24 h of incubation. The patients were treated initially for 6 weeks with four drugs regimen (clarithromycin 500 mg 12 hourly, ciprofloxacin 500 mg 12 hourly, linezolid 400 mg 12 hourly, and amikacin 500 mg 12 hourly), followed by 5 months with three drugs regimen (clarithromycin 500 mg 12 hourly, ciprofloxacin 500 mg 12 hourly, and linezolid 400 mg 12 hourly) as a maintenance dose. Follow‐up was done in every case after the completion of the proposed drug regimens. Cessation of discharge occurred within 3–4 weeks after starting treatment and, the wound was also healed or was healing in most of the cases (Figures 3 and 4).

## 4 DISCUSSION

In recent years, frequently encountered NTM species in postsurgical *wound infections are M. chelonae and M. fortuitum*.^10^ *NTM are transmitted through* aerosol, soil, dust, or contaminated tap water.^11^ In our study, all the postoperative wounds were healed initially within 7–10 days of surgery. Then within the next 1–2 months, incision sites became erythematous, and indurated, small blisters formed, burst out, and started serous discharge in small quantities. Several antibiotics were recommended for these wound‐infected cases but did not respond to any of them, discharge continued and persisted for a long time before they were referred to the microbiology department. Wound infections due to NTM usually do not occur as an early postoperative complication. During operation, wounds are contaminated with NTM from environmental sources and take some time to make their clinical appearance. After infection with NTM, the operation scar breaks down and develops a nonhealing superficial ulcer with the sinus tract from which non‐purulent serous discharge comes out.^12^

Bhalla et al.^13^ reported that 10.9% of postoperative wound infections occurred by NTM in South India. Development of mild fever, small indurations, with or without mild local pain, and serous discharge from a tiny opening of postoperative healed wound scar indicates the initiation of the onset of NTM infection. Specimens from such cases usually show no pus cell or organism on Gram stain and cultures show no growth on routine culture media for aerobic and anaerobic organisms. Hence, all these specimens should be collected through aseptic precautions, must be stained by the Z‐N staining method for acid‐fast bacilli (AFB), and incubated at 37°C after inoculation on LJ media and blood agar media to isolate NTM.^14^ The aim of accurate and early diagnosis is to formulate an appropriate treatment regimen that is specific to NTM species.

Nontuberculous mycobacteria have commonly identified pathogens from patients having postoperative wound infections and require a high suspicion for correct and early diagnosis.^13^ Chronic discharge with a prolonged course of expensive antibiotics makes it a serious type of nosocomial infection. Skin or soft tissue infection is the most common manifestation seen in NTM‐infected individuals whose wounds may be exposed directly or indirectly to the soil, colonized tap water, unsterilized operative instruments, or medical devices contaminated with environmental NTM after traumatic injury, during surgery, or cosmetic procedures. Surveillance study of environmental culture from tap water, operation instruments, wall, floor, basin, and operation theater (OT) may not yield growth of NTM.^11^ Strict sterilization of all OT equipment, proper hand washing, and prevention of wound contamination with dust, soil, and tap water are needed to prevent wound infections with NTM.

Because of the long duration of treatment, side effects, and cost of the drugs accurate diagnosis is necessary. The most preferred choice is a varying combination of antibacterial drugs like imipenem, amikacin, fluoroquinolones, doxycycline, linezolid, and clarithromycin.^15^ However, first‐line antitubercular drugs like ethambutol and rifampicin have a bactericidal effect against NTM, but they are not used commonly. However, in this series, one patient was treated with first line four drugs anti‐tuberculosis regimen for 6 months without improvement.

When there is a sudden increase in such cases, it is urgent to conduct epidemiological studies, collect specimens from the surrounding environment, and confirmation of NTM infections through culture, PCR, and antimicrobial susceptibility testing are needed. Therefore, although it is not possible to test for NTM in all patients routinely in developing countries like Bangladesh, it is advisable to refer cases to appropriate centre, if the patients had an operation, were exposed to contaminated water, dust, or soil, and suffered from discharging postoperative wounds with delayed recovery to detect whether it is NTM infection or not. Furthermore, in this background, there is poor communication between clinicians and the laboratory due to lack of awareness, unknown prevalence patterns due to lack of study, and the absence of standard diagnostic facilities and regimens for treatment.

Recently, postsurgical NTM wound infections are gradually increasing in Bangladesh. The exact cause is not known but the probable cause might be sterilization failure of instruments because some departments need different types of instruments for operation, and some instruments usually come from outside of the hospital just before operative procedure which always may not be properly sterilized. In addition, some operations need longer time, some need shunt and some need drainage tube with external opening to come out the internal collections which may be contaminated with water or soil.

Until now, there has been a lack of data regarding prevalence, diagnostic methods and treatment of NTM infections in Bangladesh. Therefore, such cases have been treated initially as general wound infections and sometimes by anti‐MTB regimens given without any diagnosis. Recently, NTM infections have attracted more attention from clinicians due to the increase in such cases; but still, there is a lack of awareness. When persistent chronic discharge from postoperative wound infections occur after operations that cannot be cured by usual antibiotics, NTM infections should be suspected and Z‐N stain, culture, Gene‐Xpert, and PCR must be considered as diagnostic tools to treat the patients with appropriate anti‐NTM drug regimen.

## AUTHOR CONTRIBUTIONS

**Kakali Halder:** Conceptualization; formal analysis; project administration; writing – original draft; writing – review and editing. **Nusrat Noor Tanni:** Formal analysis; investigation; methodology; writing – review and editing. **Rubaiya Binte Kabir:** Formal analysis; investigation; methodology; resources. **Maherun Nesa:** Formal analysis; methodology; resources; writing – review and editing. **Md. Faizur Rahman:** Investigation; methodology; resources. **Rizwana Zaman:** Data curation; investigation. **Farjana Binte Habib:** Formal analysis; investigation; methodology. **Noor‐E‐Jannat:** Investigation; methodology; resources. **Md. Asaduzzaman:** Investigation; resources; software. **Azmeri Haque:** Investigation; resources. **Akteruzzaman Chowdhury:** Investigation; methodology; resources. **Avizit Sarker:** Formal analysis; investigation. **Nadira Akter:** Investigation; methodology; resources. **Mahbuba Chowdhury:** Methodology; resources; writing – review and editing. **Sazzad Bin Shahid:** Funding acquisition; resources; writing – review and editing. **S.M. Shamsuzzaman:** Conceptualization; funding acquisition; project administration; supervision; validation; visualization; writing – review and editing.

## FUNDING INFORMATION

Self funded.

## CONFLICT OF INTEREST STATEMENT

None.

## CONSENT

Informed written consent was taken from all patients.

## Data Availability

The data that supports the findings of the study are available from corresponding author upon request.
